# The Use of Juçara (*Euterpe edulis* Mart.) Supplementation for Suppression of NF-κB Pathway in the Hypothalamus after High-Fat Diet in Wistar Rats

**DOI:** 10.3390/molecules23071814

**Published:** 2018-07-21

**Authors:** Aline Boveto Santamarina, Giovana Jamar, Laís Vales Mennitti, Veridiana Vera de Rosso, Helena Cassia Cesar, Lila Missae Oyama, Luciana Pellegrini Pisani

**Affiliations:** 1 Programa de Pós-Graduação Interdisciplinar em Ciências da Saúde, Universidade Federal de São Paulo, São Paulo 11015-020, Brazil; alinesantamarina@gmail.com (A.B.S.); gi.jamar@gmail.com (G.J.); laisvmennitti@hotmail.com (L.V.M.); helenacesarbr@gmail.com (H.C.C.); 2 Departamento de Biociências, Universidade Federal de São Paulo, São Paulo 11015-020, Brazil; veriderosso@yahoo.com; 3 Departamento de Fisiologia, Universidade Federal de São Paulo, São Paulo 04023-062, Brazil; lmoyama@gmail.com; 4 Laboratório de Nutrição e Fisiologia Endócrina (LaNFE); Departamento de Biociências, Instituto de Saúde e Sociedade, Universidade Federal de São Paulo, Rua Silva Jardim, 136, Térreo, Vila Mathias, Santos, São Paulo 11015-020, Brazil

**Keywords:** juçara, inflammation, hypothalamus, short-term high-fat diet, NF-κB pathway

## Abstract

Obesity is associated with modern diets that are rich in saturated fatty acids. These dietary patterns are linked to low-grade proinflammatory mechanisms, such as the toll-like receptor 4/nuclear factor kappa-B (NF-κB) pathway rapidly activated through high-fat diets. Juçara is a berry rich in anthocyanins and unsaturated fatty acids, which prevents obesity and associated comorbidities. We evaluated the effect of different doses of freeze-dried juçara pulp on NF-κB pathway after the consumption of short-term high-fat diet. Male Wistar rats with *ad libitum* access to food and water were divided into four groups: Control diet (C), high-fat diet (HFC), high-fat diet with 0.25% juçara (HFJ 0.25%), and high-fat diet with 0.5% juçara (HFJ 0.5%). Energy intake and body weight gain were increased in HFC and HFJ 0.5% groups compared to C group. The hypothalamus weight reduced in the HFC group compared to C and HFJ 0.25% groups. Cytokines, MYD88, TRAF6, and pNF-κBp50 levels in the hypothalamus, serum triacylglycerol, LDL-cholesterol (LDL-C), and free fatty acid levels were improved in the HFJ 0.25% group. In summary, the HFJ 0.25% group had better protective effects than those in the HFJ 0.5%. Therefore, 0.25% juçara can be used to protect against central inflammation through the high-fat diet-induced NF-κB pathway.

## 1. Introduction

In obesity, deleterious mechanisms are activated early, even before significant weight gain occurs [1]. Dietary patterns have drastically changed, and the modern diets are mainly based on processed foods [2]. The convenience of obtaining processed foods has promoted the increased consumption of high-fat diets, which are rich in saturated fatty acids (SFAs) and sugars and poor in dietary fibers [1,3]. This unhealthy dietary practice can rapidly induce asymptomatic and deleterious metabolic mechanisms, favoring the development of obesity-related chronic diseases, such as metabolic syndrome and cardiovascular disease [4].

Intake of a high-fat diet containing SFAs results in low-grade inflammation both in vitro and in vivo, which is mediated by the toll-like receptor 4 (TLR4). TLR4 recognizes not only lipid ligands from pathogens, such as bacteria, but also SFAs, which ultimately results in the activation of the nuclear factor kappa-B (NF-κB), promoting an increase in chronic subclinical inflammation and playing an important signaling role in the pathogenesis of noninfectious inflammatory and chronic noncommunicable diseases [5].

The inflammatory process mediated by SFA intake has considerable effects on the central nervous system (CNS), mainly the hypothalamus. SFAs exert deleterious effects on the anatomy and physiology of the hypothalamus, which is responsible for performing several functions, such as energy homeostasis [6]. Thaler & Schwartz [7] reported that the hypothalamus is the first target of inflammation in high-fat diet-induced obesity and may be the origin of obesity-related symptoms even before other peripheral organs are affected. In addition, hypothalamic inflammation leads to unbalanced leptin and insulin signaling, contributing to the development of obesity.

On the contrary, polyunsaturated fatty acids (PUFAs) ω-3 and ω-6 have shown high potential in inhibiting the NF-κB inflammatory response mediated by TLR4, thus representing a nutritional strategy for preventing and treating obesity and its comorbidities [5]. Anthocyanins are also known for their powerful antioxidant properties and health benefits. These red or purple plant pigments of the flavonoid class possess arteriosclerotic, antiobesity, and antidiabetic properties [8,9].

Tropical countries, such as Brazil, have abundant biodiversity and untapped resources of native fruits which potentially possess health benefits [10]. Juçara (*Euterpe edulis* Mart.) is a native Atlantic Rainforest palm tree fruit, which produces a dark purple creamy pulp that has a high nutritional value [11,12]. Considering the similarity in the pulp composition and phylogenetic parentage between the açai (*Euterpe oleracea* Mart.) and juçara palm tree, we speculated that the juçara fruit pulp could have similar beneficial effects as the açai fruit pulp for treating several diseases [12,13]. Juçara pulp has been shown to be effective for treating several diseases [13]. It contains significant amounts of dietary fibers, monounsaturated fatty acids (MUFAs), and PUFAs. It also contains high levels of flavonoids, such as anthocyanins, and phenolic compounds [14,15].

Few studies have addressed the effect of the intake of different doses of bioactive food compounds and short-term high-fat diets on inflammation and metabolism [16,17]. Considering the lack of research in this field, the present study aimed to evaluate the effectiveness of different doses of juçara pulp in preventing the deleterious effects of a short-term high-fat diet on the modulation of the NF-κB pathway. For this purpose, we performed four experimental groups: control standard chow (C); high-fat diet control (HFC); high-fat diet with 0.25% freeze-dried juçara pulp (HFJ 0.25%), and high-fat diet with 0.5% freeze-dried juçara pulp (HFJ 0.5%) during 7-days.

## 2. Results

### 2.1. Energy Intake, Body and Tissue Weights

**Figure 1:**
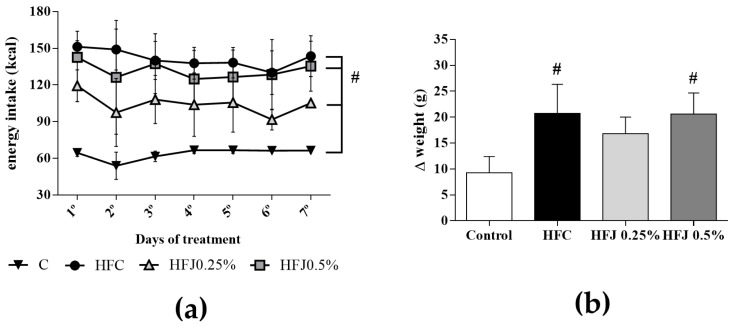
(**a**) Mean daily energy intake estimative during the experiment; (**b**) body weight gain (Δ). # *p* < 0.05 compared with the control diet (C) group (*n* = 6 or 7).

Energy intake in all the experimental diet groups (HFC, HFJ 0.5%, and HFJ 0.25%) was higher than that in the C group since the first day of the treatment probably due to the higher energy density of the experimental diets (*p* ≤ 0.043; Figure 1a). Compared with the C group, body weight gain (Δ) was increased in the HFC (*p* = 0.032) and HFJ 0.5% (*p* = 0.042) groups; however, there was no difference in body weight gain in the HFJ 0.25% group (Figure 1b).

Additionally, hypothalamic mass reduced in the HFC group compared to the C (*p* = 0.028) and HFJ 0.25% (*p* = 0.047) groups. The relative weight of retroperitoneal adipose tissue (RET) and sum of visceral white adipose tissues (ΣWAT) in the HFC group were significantly higher than those in the C group (*p* = 0.041 and *p* = 0.029, respectively). In addition, the relative tissue weights of the liver, epididymal adipose tissue (EPI), and mesenteric adipose tissue (MES) were not different among the groups (Table 1).

### 2.2. Lipoproteins, Triacylglycerol, Free Fatty Acids and Adiponectin

The HFC group demonstrated higher serum total cholesterol (TC; *p* = 0.021) and LDL-C (*p* = 0.047) levels than those in the C group. On the contrary, the HFJ 0.25% group showed lower serum free fatty acid (FFA; *p* = 0.034) and LDL-C (*p* = 0.050) levels than those in the HFC group. The HFJ 0.25% group also showed higher serum adiponectin levels than those in the C group. The HFJ 0.5% groups showed higher levels of triacylglycerol (TAG) than those in the HFJ 0.25% (*p* = 0.049) and C (*p* = 0.021) groups. HDL-C levels did not differ among the groups (Table 2).

### 2.3. Hypothalamic Cytokine Concentration

**Figure 2:**
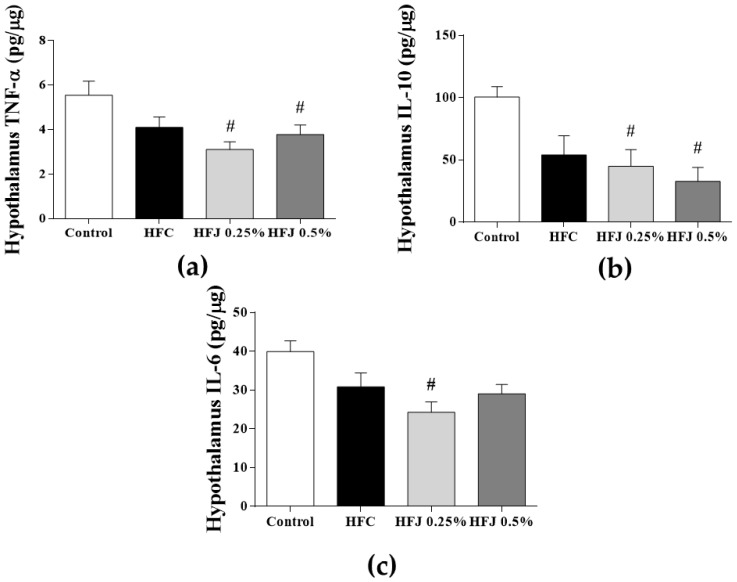
Cytokine levels of protein extract samples from the hypothalamus: (**a**) TNF-α, (**b**) Il-10, and (**c**) IL-6. * *p* < 0.05 compared with the high-fat juçara 0.25% (HFJ 0.25%) group; # *p* < 0.05 compared with the control diet (C) group (*n* = 6 or 7).

Tumor necrosis factor-α (TNF-α) levels in the protein extract samples from the hypothalamus were significantly lower in the HFJ 0.25% (*p* = 0.029) and HFJ 0.5% (*p* = 0.041) groups than in the C group. Compared with the C group, the hypothalamic IL-6 levels were reduced in the HFJ 0.25% group (*p* = 0.007). Furthermore, hypothalamic IL-10 levels were increased in the HFJ 0.25% and HFJ 0.5% groups (*p* = 0.016 and *p* = 0.018, respectively) compared with the control group (Figure 2).

### 2.4. Hypothalamic NF-κB Pathway Protein Expression

**Figure 3:**
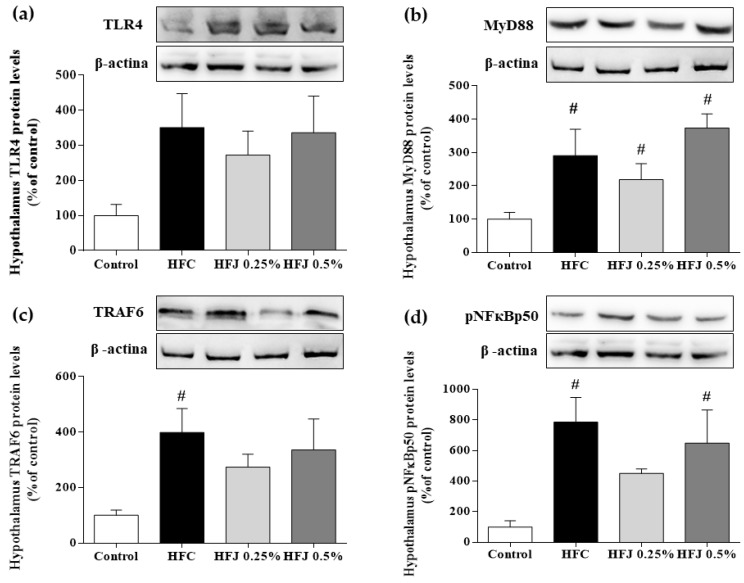
Protein expression in the NF-κB pathway in the hypothalamus: (**a**) TLR4, (**b**) MYD88, (**c**) TRAF6, and (**d**) pNF-κBp50. The housekeeping used for all analyses was β-actin expression. * *p* < 0.05 compared with the high-fat juçara 0.25% (HFJ 0.25%) group; # *p* < 0.05 compared with the control diet (C) group (*n* = 6).

Although no difference was found in the protein expression of the TLR4 membrane receptor in the hypothalamus, a significant increase was found in the myeloid differentiation primary response gene 88 (MYD88) expression in the HFC, HFJ 0.25%, and HFJ 0.5% groups (*p* < 0.001, *p* = 0.003, and *p* = 0.004, respectively) compared with that in the C group. Compared with the C group, there was a significant increase in the TNF receptor-associated factor 6 (TRAF6) expression in the HFC group (*p* = 0.016). The phosphorylation of the nuclear transcription factor NF-κBp50 subunit in the HFC and HFJ 0.5% groups was higher than that in the C group (*p* = 0.016 and *p* = 0.032, respectively; Figure 3).

## 3. Discussion

We elucidated the effects of supplementation of different doses of juçara pulp (0.5% and 0.25%) on metabolic modulation and the NF-κB inflammatory pathway in the hypothalamus after consumption of a short-term high-fat diet. The lower dose of juçara pulp was clearly more effective than the higher dose with respect to preventing the hypothalamic inflammatory process.

Energy intake was significantly higher in all high-fat diet groups (HFC, HFJ 0.25%, and HFJ 0.5%) due to the higher energy density of the diets than that in the C group. Body weight gain (Δ) in the HFC group was increased as expected, indicating that the high-fat diet model was efficient in inducing body weight gain even with short-term diet intake [18,19]. Interestingly, the body weight of rats in the HFJ 0.25% group did not differ from that in the C group, suggesting that low doses of juçara have a protective role against body weight gain and consequently obesity development. The beneficial role of juçara has previously been reported with the consumption of juçara with high- [20] and normal-fat diets [15], which could be attributed to the nutritional components of the fruit.

White adipose tissue (WAT) is well known for its metabolic and endocrine functions, and our results showed an increase in adipose depots as RET (retroperitoneal adipose tissue) and ΣWAT (sum of visceral white adipose tissues), which were demonstrated in the HFC group. Nevertheless, the concomitant consumption of juçara with high-fat diets inhibited the gain of visceral adiposity, which contributed to homeostatic maintenance compared with the control group. Studies on bioactive compound and unsaturated fatty acid supplementation have demonstrated a reduction in adiposity, leading to an improvement in metabolic functions [21,22].

Adiponectin is an adipokine exclusively secreted by the adipose tissue; it has cardioprotective, anti-inflammatory, and antiatherosclerotic properties. Furthermore, it plays a role as an insulin sensitizer, thereby stimulating glucose uptake and lipid oxidation in muscles and inhibiting gluconeogenesis [23]. In obesity, adiponectin levels and its membrane receptor expression are generally reduced [23]. The increase in adiponectin levels in the HFJ 0.25% group rather than in the HFJ 0.5% and HFC groups clarified that the anti-inflammatory effect of juçara pulp is dose-specific. Consistently, IL-6 and TNF-α levels in the hypothalamus were reduced in the HFJ 0.25% group.

Exposure to a high-fat diet rich in SFAs can reduce the neuronal score in animal models through the initiation of the apoptotic pathway in the hypothalamus, resulting in the alteration of brain structure, function, and dimension [24]. We found a significant difference in absolute and relative hypothalamic weights among the groups, suggesting that high-fat diet intake induced these mechanisms. In contrast, juçara supplementation inhibited these deleterious effects of high-fat diets in the hypothalamus. The effect of juçara on the central nervous system has not been described, but evidence has shown that PUFAs and polyphenols play neuroprotective roles. Hence, considering juçara composition, we believe that it also exerts neuroprotective effects [25,26].

After exposure to a large amount of dietary fats for 1 day, the hypothalamus is the first target of inflammation and is the first tissue to be affected. A substantial increase was observed in hypothalamic inflammation in a short-term after high-fat diet intake, and inflammation intensity was blunted after 2–3 weeks but reversed after 3–4 weeks [27]. This proinflammatory effect of a high-fat diet leads to unbalanced leptin and insulin anorexigenic signaling in the hypothalamus due to local inflammatory activation-associated mechanisms [6]. We observed a reduction in the inflammatory pathway in the HFJ 0.25% group that could be attributed to the protective action of the bioactive compounds of juçara against the damage caused by high-fat diet intake.

An extensive review of the literature showed a strong relationship between the TLR4 pathway and high-fat diet intake in metabolic disorders. The major function of TLR4 is to recognize pathogen-associated molecular patterns as lipopolysaccharides (LPS) present in the membrane of gram-negative bacteria. Nevertheless, it may also be stimulated by the presence of SFAs in the diet, leading to a proinflammatory response [1,5]. In the short term, the hypothalamic tissue was described as the main site of the TLR4 pathway activity due to increased signaling proteins involved in NF-κB phosphorylation, which promotes gene transcription of proinflammatory cytokines [7,28]. Our data provided evidence on reduced phosphorylation of the NF-κBp50 subunit that was stimulated by the addition of 0.25% juçara in the diet. Although we did not observe any reduction in the amount of TLR4 protein, our findings showed that pNF-κBp50 alterations were mediated by TLR4 activity through the MYD88-dependent pathway, as noted by the concomitant reduction in the amount of MYD88 and TRAF-6 proteins. These results suggested that 0.25% of juçara inhibited the inflammatory response by reducing NF-κB phosphorylation despite evident proinflammatory stimulus of a high-fat diet intake. This hypothesis was confirmed by reduced cytokine levels in the hypothalamus, showing decreased activity of the NF-κB pathway.

Considering the composition of juçara pulp, which is abundant in anthocyanins, MUFAs, and PUFAs, we postulated that the anti-inflammatory effect of juçara reaches CNS at low doses (0.25%). Our hypothesis is supported by the result of previous studies showing that the consumption of MUFAs and PUFAs can reverse hypothalamic inflammation [26,29,30]. Moreover, despite the lack of information regarding the effect of anthocyanins on the hypothalamus, studies demonstrating their positive effect on other tissues indicated that anthocyanins also have an anti-inflammatory activity in CNS [31,32]. Based on this finding, we believe that the nutritional composition of juçara can provide a strong anti-inflammatory action even at small doses.

Notably, the HFJ 0.5% group did not demonstrate greater efficiency in the modulation of cytokines involved in the inflammatory profile of obesity and was maintained at a level similar to that of the HFC group. We hypothesized that this result is associated with increased energy intake, whereas the amounts of calories and fatty acids from the pulp were higher. Studies have shown that even PUFAs and MUFAs have deleterious effects when consumed in substantial amounts [33,34]. This highlights the importance of considering not only the quality of fatty acids present in the diet but also their quantity which plays a major role in metabolic and inflammatory outcomes.

Serum analyses showed that juçara supplementation improved the lipid profile despite high-fat diet intake. Accordingly, our results showed that the HFJ 0.25% group showed better biological activity and exhibited improvement in TAG and LDL-C levels than in the HFC and HFJ 0.5% groups, respectively. Thus, the superiority of the 0.25% dose of juçara could be confirmed by previous studies that used 0.5% and 2% of juçara pulp, which were associated with a hypercaloric diet and did not show any improvement in the lipid profile [20,35]. In addition, the HFJ 0.25% group showed effective reduction of serum FFA levels, which is a remarkable finding because high FFA levels are related to insulin resistance, cellular apoptosis, and proinflammatory cytokine production. These results reaffirmed the protective and anti-inflammatory activities of juçara [36,37]. These findings also indicated that positive metabolic effects can be expected in the HFJ 0.25% group. These effects can be largely attributed to the optimal amount of fatty acid composition of the fruit pulp which is abundant in MUFAs and PUFAs that have the potential to improve TAG and LDL-C levels. These fatty acids attenuate risk factors related to obesity and particularly contribute to the improvement in insulin sensitivity, blood pressure, endothelial dysfunction, and subclinical inflammatory parameters [11,38].

A trial using two doses of chokeberry (100 and 200 mg/kg/day), similar to those used in our study, improved the lipid profile in the experimental group compared with that in the control group, but no differences were observed between the doses [39]. Likewise, studies on diet supplementation with fruits rich in anthocyanins showed similar results. A study on mice with 5–10% bilberry extracts added to high-fat chow showed improvement in the inflammatory response, but no differences were observed between the doses. A test using jaboticaba peel freeze-dried powder at 1%, 2%, and 4% showed a reduction in IL-6, IL1β, and IKBα levels to those in the control group, but no differences were found among the doses [31,40].

This showed that compared with using controls, using different doses of anthocyanins could reverse the negative effects of obesity. Nevertheless, higher doses did not have a more pronounced or better effect on inflammatory and metabolic parameters. Hence, large doses of bioactive compounds are not required to have beneficial effects, and nonphysiological doses should be carefully used, with the intention of obtaining a stronger effect.

It is important to note that foods should be considered in terms of their complete composition and not by individual nutrients. Besides, the importance of the effect of dose on animals’ health should be emphasized. In this sense, diet containing 0.25% of juçara can be considered as a potent nutraceutical for treating chronic metabolic disorders related to chronic subclinical inflammation.

Taken together, low-dose juçara can be an excellent food for treating and preventing metabolic disorders associated with high-fat diet intake. Nevertheless, further investigations must be conducted to clarify its effects on other populations and different treatment periods; the role of all nutrients involved in the beneficial effects of juçara intake should also be clarified.

## 4. Materials and Methods

### 4.1. Freeze-Dried Juçara Pulp Powder

Juçara pulp (*E. edulis* Mart.) was obtained from the agroecological Juçara Project—Instituto de Permacultura e Ecovilas da Mata Atlântica (Ubatuba, SP, Brazil) and freeze-dried. Nutritional characterization and bioactive compound composition of juçara pulp are previously described [14,15,41] and are presented in Table 3.

Intake of 100.5–350.0 mg anthocyanin per day is considered safe and has been shown to improve lipid profiles and inflammatory responses [42,43]. We considered 50 g of chow/day/rat as the mean of diet intake for calculation purposes. The proportional human consumption using allometric factors proposed by the Food and Drug Administration in 2005 [44] showed that a 0.5% dose corresponded to 3.3 mg of anthocyanin/kg/day, which could be obtained by consuming 100 g of fresh juçara pulp or 10 g of lyophilized juçara per day by an adult with an average weight of 70 kg. The second dose (0.25%) was determined by offering half of the first dose, representing 50 g of fresh juçara pulp or 5 g of lyophilized juçara per day for a 70-kg adult to evaluate the effectiveness of low doses in the inflammatory process. The average daily intake of anthocyanins using the upper dose (0.5%) was 6 mg/rat/day, corresponding to physiological and nonpharmacological doses [45].

### 4.2. Animal Treatment

All animal experiments were performed according to the protocols approved by the Experimental Research Committee of the Universidade Federal de São Paulo (CEUA nº 5252010715). We followed the standards of the 2013 Brazilian Guidelines for Care and Use of Animals for Scientific Purposes and Teaching issued by the National Council of Animal Experimentation Control [46]. A total of 27 outbred male Wistar rats aged 90 days were used.

The animals were maintained in collective polypropylene cages in an isolated room with controlled temperature (23 °C ± 2 °C), humidity (60% ± 5%), and lighting (12-h light/dark cycle). They received food and water ad libitum for the whole experimental period of 7 days. After 1 week of acclimatization, the animals were randomly divided into four experimental groups: control standard chow (C; *n* = 6); high-fat diet control (HFC; *n* = 7); high-fat diet with 0.25% juçara (HFJ 0.25%; *n* = 7), and high-fat diet with 0.5% juçara (HFJ 0.5%; *n* = 7). The animals received their respective diets ad libitum, and diet consumption was measured to assess the calorie intake. The control group (C) received a rodent standard commercial diet, the high-fat diet model used was stablished by Dornellas [18] whereupon the main ingredient is the standard commercial diet with changes in the amount of fat. Juçara supplementation was done by mixing the powdered freeze-dried juçara pulp in the modified diet before pelleting. All experimental diets were stored at −20 °C and protected from light. The detailed composition of the ingredients used in the diets preparation, as well as, the distribution of macronutrients and energy values are described in Table 4.

To estimate the mean calorie diet intake, we used the energy values of each diet provide in Table 4 and the animals chow intake weighed every day during the experimental period. To access the obesogenic effectiveness of the diet we evaluated the body mass gain after the 7-days experimental procedure. We weighted all animals at the first day and last day of experimental procedure to obtain the Δ values (Δ = last day body weight − first day body weight) which reflect the difference of body mass before and after the treatment

At the end of the experimental period, the animals were anesthetized using ketamine (80 mg/kg) and xylazine (10 mg/kg) and euthanized by decapitation in the morning between 08:00 and 10:00 after 12-h fasting. Blood was collected and hypothalamus dissected from other brain structures by a trained person for analyses and were immediately stored at −80 °C. All experimental procedures were performed using single samples, and sample pooling was not required. The liver was weighed, retroperitoneal adipose tissue (RET), epididymal adipose tissue (EPI), and mesenteric adipose tissue (MES) were collected and weighed to calculate the sum of visceral white adipose tissue (ΣWAT).

### 4.3. Serum Parameters

Serum glucose, total cholesterol (TC), HDL-cholesterol (HDL-C), and triacylglycerol (TAG) levels were analyzed by colorimetric method using commercial kits (Labtest, Lagoa Santa, MG, Brazil). FFA analysis was performed using a colorimetric kit (Zen-bio Inc., Research Triangle Park, Durham, AC, USA) according to the manufacturer’s instructions. Adiponectin concentration was measured using ELISA kits (DuoSet ELISA, R&D Systems; Minneapolis, MN, USA), and serum concentrations of LDL-C were estimated indirectly using the Friedewald equation: LDL-C = total cholesterol (HDL-C) − (TAG/5) [47].

### 4.4. Tissue Cytokine Concentrations

Hypothalamus samples were homogenized in a specific buffer (containing 100 mM Tris-HCl (pH 7.5), 1% Triton X-100, 10% sodium dodecyl sulfate (SDS), 10 mM EDTA, 100 mM sodium fluoride, 10 mM sodium pyrophosphate, 10 mM sodium orthovanadate, 2 mM phenylmethylsulphonyl fluoride, and 0.1 mg aprotinin from bovine lung/mL.) and centrifuged at 20,800× *g* for 40 min at 4 °C. The supernatant was stored and used for the measurement of TNF-α, IL-6, and IL-10 concentrations using commercial ELISA kits (DuoSet ELISA, R&D Systems, Minneapolis, MN, USA) according to the manufacturer’s instructions.

### 4.5. Western Blot Analyses

Hypothalamus samples were homogenized in lysis buffer containing 100 mM Tris-HCl (pH 7.5), 1% Triton X-100, 10% sodium dodecyl sulfate (SDS), 10 mM EDTA, 100 mM sodium fluoride, 10 mM sodium pyrophosphate, 10 mM sodium orthovanadate, 2 mM phenylmethylsulphonyl fluoride, and 0.1 mg aprotinin from bovine lung/mL. The homogenized sample was centrifuged at 20,800× *g* for 40 min at 4 °C, and the supernatant was collected. The total protein concentration was measured using Bradford reagent (LGC Laboratories, Inc., Cotia, SP, Brazil).

Protein samples were separated by electrophoresis on a 10% SDS polyacrylamide gel and transferred to nitrocellulose membranes. Then, 1% bovine serum albumin solution was used to block the membranes overnight at room temperature. The membranes were incubated overnight with the following primary antibodies: pNF-κBp50 (sc-101744; Santa Cruz Biotechnology, Inc., Santa Cruz, CA, USA) and TLR4 (ab22048), MYD88 (ab2064), TRAF6 (ab33915), and β-actin (ab6276; AbCam, Cambridge, UK). Membranes were incubated with horseradish peroxidase-conjugated secondary antibodies for 1 h at room temperature.

Bands were visualized using enhanced chemiluminescence scanned using UVITec (Cambridge, UK) after adding ECL reagent (Bio-Rad Laboratories, Inc, Hercules, CA, USA). The intensity of each band was quantified by ImageJ software (ImageJ, National Institute of Health, Bethesda, MD, USA). Calculations for each band obtained from the analysis of the protein of interest were normalized to β-actin levels.

### 4.6. Statistical Analyses

Initially, the Grubb’s test was performed to eliminate significant outliers. For variation in energy intake during the treatment, we performed ANOVA for repeated measures, followed by the Bonferroni post hoc test. To verify the interfaces among groups, we used the one-way ANOVA, followed by the Bonferroni post hoc test. The level of significance was set at *p* ≤ 0.05. The descriptive analysis was demonstrated using the mean ± S.E.M. Statistical analysis was performed using the SPSS version 22.0 software. The sample size was chosen based on the guidelines established by the National Animal Research Ethics Committee and previous research conducted by our group, ensuring the necessary numbers to maintain strength and consistency of statistical analyses [15,35,46,48,49].

## Figures and Tables

**Table 1:** Relative tissue weight among different experimental groups.

Tissue	(g/100 g of Body Mass)							
	Control (*n* = 6)		HFC (*n* = 7)		HFJ 0.25% (*n* = 7)		HFJ 0.5% (*n* = 7)	
	Mean	S.E.M.	Mean	S.E.M.	Mean	S.E.M.	Mean	S.E.M.
Hypothalamus	0.03	0.003	0.02 ^#,^*	0.001	0.03	0.002	0.03	0.001
Liver	2.92	0.09	2.81	0.05	2.87	0.07	2.80	0.03
RET	0.93	0.13	1.68 ^#^	0.14	1.27	0.14	1.39	0.15
EPI	1.03	0.085	1.37	0.112	1.16	0.12	1.06	0.14
MES	0.98	0.16	1.03	0.10	0.96	0.03	1.09	0.09
ΣWAT	2.94	0.32	4.07 ^#^	0.21	3.39	0.26	3.54	0.30

^#^*p* < 0.05 versus control group (C) (*n* = 6 or 7); * *p* < 0.05 versus to high-fat juçara 0.25% (HFJ 0.25%) (*n* = 6 or 7).

**Table 2:** Lipoproteins, triacylglycerol, free fatty acids and adiponectin analyses performed among the different experimental groups.

Parameter	Experimental Groups							
	Control (*n* = 6)		HFC (*n* = 7)		HFJ 0.25% (*n* = 7)		HFJ 0.5% (*n* = 7)	
	Mean	S.E.M	Mean	S.E.M.	Mean	S.E.M.	Mean	S.E.M.
triacylglycerol (mg/dL)	104.84	2.91	117.96	7.15	109.84	4.86	120.55 *^,#^	6.70
total cholesterol (mg/dL)	108.13	6.24	136.40 ^#^	10.44	118.04	6.02	133.41	12.38
HDL-Cholesterol (mg/dL)	54.72	3.44	46.21	4.05	48.86	3.96	47.11	4.30
LDL-Cholesterol (mg/dL)	41.30	9.49	68.32 ^#,^*	5.15	50.79	9.38	58.42	9.95
Free fatty acids (mM/mL)	1.87	1.65	2.05 *	2.74	1.39	5.40	1.97	2.08
Adiponectin (ng/mL)	1.77	0.029	1.92	0.33	2.69	0.48 ^#^	1.95	0.32

^#^*p* < 0.05 versus control group (C); * *p* < 0.05 versus to high-fat juçara 0.25% (HFJ 0.25%).

**Table 3:** Juçara pulp (*E. edulis* Mart.) characterization.

Juçara Pulp	Concentration in 100 g of Fresh Matter		References
	Mean	S.E.M	
Moisture (%)	88.7	3.8	[14]
*Carbohydrates* (g)	28.3	3.5	[14]
Proteins (g)	6.0	0.3	[14]
Lipids (g)	29.2	0.9	[14]
*Palmitic acid (%)*	34.43	3.42	[41]
*Stearic acid (%)*	3.01	0.30	[41]
SAT (%)	37.44		[41]
*Palmitoleic acid (%)*	2.61	0.26	[41]
*Oleic acid (%)*	35.96	3.08	[41]
MUFA (%)	38.57		[41]
*Linoleic acid (%)*	19.18	1.89	[41]
*Linolenic acid (%)*	0.91	0.20	[41]
PUFA (%)	20.08		[41]
Fiber (g)	28.3	0.3	[14]
Ashes (g)	8.8	0.8	[14]
*Energetic values (kcal)*	*400.0*	*23.9*	[14]
Cyanidin 3-rutinoside (mg)	191.0	6.5	[15]
Cyanidin 3-glucoside (mg)	71.4	2.1	[15]
*Total anthocyanins (mg)*	*262.4*	*8.6*	[15]
Apigenin deoxyhexosyl-hexoside (mg)	25.4	1.5	[14]
Luteolin deoxyhexosyl-hexoside (mg)	37.6	1.9	[14]
Dihydrokaempferol-hexoside (mg)	66.4	2.6	[14]
*Total phenolic compounds (mg)*	*415.1*	*22.3*	[14]

**Table 4:** Ingredients and nutritional composition of experimental diets proposed by Dornellas [18] supplemented or not with juçara pulp.

Ingredients	Diet (g/100 g)			
	Control	HF Control	HF Juçara 0.25%	HF Juçara 0.5%
Standard chow	100	50	50	50
Sucrose	-	10	10	10
Casein	-	20	20	20
Soybean Oil	-	2	2	2
Lard	-	18	18	18
Butyl hydroquinone	-	0.004	0.004	0.004
Juçara pulp powder	-	-	0.25	0.5
Protein	22.4	23.6	23.6	23.6
Total fat	4.8	22	22	22
Carbohydrates	39.1	26.8	26.8	26.8
Alimentary fiber	11.4	15.1	15.1	15.1
Mineral residues	11.9	9	9	9
Energy (Kcal/100 g)	270	410	420	430

## References

[1] Myles I.A. (2014). Fast food fever: Reviewing the impacts of the Western diet on immunity. Nutr. J..

[2] Zobel E.H., Hansen T.W., Rossing P., von Scholten B.J. (2016). Global changes in food supply and the obesity epidemic. Curr. Obes. Rep..

[3] Swinburn B., Caterson I., Seidell J., James W. (2007). Diet, nutrition and the prevention of excess weight gain and obesity. Public Health Nutr..

[4] Turner N., Kowalski G.M., Leslie S.J., Risis S., Yang C., Lee-Young R.S., Babb J.R., Meikle P.J., Lancaster G.I., Henstridge D.C. (2013). Distinct patterns of tissue-specific lipid accumulation during the induction of insulin resistance in mice by high-fat feeding. Diabetologia.

[5] Rogero M.M., Calder P.C. (2018). Obesity, inflammation, toll-like receptor 4 and fatty acids. Nutrients.

[6] Namavar M.R., Raminfard S., Jahromi Z.V., Azari H. (2012). Effects of high-fat diet on the numerical density and number of neuronal cells and the volume of the mouse hypothalamus: A stereological study. Anat. Cell Biol..

[7] Thaler J.P., Schwartz M.W. (2010). Minireview: Inflammation and obesity pathogenesis: The hypothalamus heats up. Endocrinology.

[8] Li H., Jiao Y., Xie M. (2017). Paeoniflorin ameliorates atherosclerosis by suppressing TLR4-mediated NF-κB activation. Inflammation.

[9] Esposito D., Damsud T., Wilson M., Grace M.H., Strauch R., Li X., Lila M.A., Komarnytsky S. (2015). Black currant anthocyanins attenuate weight gain and improve glucose metabolism in diet-induced obese mice with intact, but not disrupted, gut microbiome. J. Agric. Food Chem..

[10] Schreckinger M.E., Lotton J., Lila M.A., de Mejia E.G. (2010). Berries from South America: A comprehensive review on chemistry, health potential, and commercialization. J. Med. Food.

[11] Da Silva Campelo Borges G., Vieira F.G.K., Copetti C., Gonzaga L.V., Zambiazi R.C., Mancini Filho J., Fett R. (2011). Chemical characterization, bioactive compounds, and antioxidant capacity of jussara (*Euterpe edulis*) fruit from the Atlantic Forest in southern Brazil. Food Res. Int..

[12] Schulz M., da Silva Campelo Borges G., Gonzaga L.V., Oliveira Costa A.C., Fett R. (2016). Juçara fruit (*Euterpe edulis* Mart.): Sustainable exploitation of a source of bioactive compounds. Food Res. Int..

[13] Felzenszwalb I., da Costa Marques M.R., Mazzei J.L., Aiub C.A. (2013). Toxicological evaluation of Euterpe edulis: A potential superfruit to be considered. Food Chem. Toxicol..

[14] da Silva N.A., Rodrigues E., Mercadante A.Z., de Rosso V.V. (2014). Phenolic compounds and carotenoids from four fruits native from the brazilian atlantic forest. J. Agric. Food Chem..

[15] Morais C.A., Oyama L.M., de Moura Conrado R., de Rosso V.V., do Nascimento C.O., Pisani L.P. (2015). Polyphenols-rich fruit in maternal diet modulates inflammatory markers and the gut microbiota and improves colonic expression of ZO-1 in offspring. Food Res. Int..

[16] Das N., Sikder K., Bhattacharjee S., Majumdar S.B., Ghosh S., Majumdar S., Dey S. (2013). Quercetin alleviates inflammation after short-term treatment in high-fat-fed mice. Food Funct..

[17] Most J., Goossens G.H., Jocken J.W.E., Blaak E.E. (2014). Short-term supplementation with a specific combination of dietary polyphenols increases energy expenditure and alters substrate metabolism in overweight subjects. Int. J. Obes..

[18] Dornellas A.P.S., Watanabe R.L.H., Pimentel G.D., Boldarine V.T., Nascimento C.M.O., Oyama L.M., Ghebremeskel K., Wang Y., Bueno A.A., Ribeiro E.B. (2015). Deleterious effects of lard-enriched diet on tissues fatty acids composition and hypothalamic insulin actions. Prostaglandins Leukot. Essent. Fat. Acids.

[19] Wiedemann M.S.F., Wueest S., Item F., Schoenle E.J., Konrad D. (2013). Adipose tissue inflammation contributes to short-term high-fat diet-induced hepatic insulin resistance. Am. J. Physiol. Endocrinol. Metab..

[20] Oyama L.M., Silva F.P., Carnier J., De Miranda D.A., Santamarina A.B., Ribeiro E.B., Oller Do Nascimento C.M., De Rosso V.V. (2016). Jucąra pulp supplementation improves glucose tolerance in mice. Diabetol. Metab. Syndr..

[21] Seymour E.M., Lewis S.K., Urcuyo-Llanes D.E., Tanone I.I., Kirakosyan A., Kaufman P.B., Bolling S.F. (2009). Regular tart cherry intake alters abdominal adiposity, adipose gene transcription, and inflammation in obesity-prone rats fed a high fat diet. J. Med. Food.

[22] Razquin C., Martinez J.A., Martinez-Gonzalez M.A., Mitjavila M.T., Estruch R., Marti A. (2009). A 3 years follow-up of a Mediterranean diet rich in virgin olive oil is associated with high plasma antioxidant capacity and reduced body weight gain. Eur. J. Clin. Nutr..

[23] Villarreal-Molina M.T., Antuna-Puente B. (2012). Adiponectin: Anti-inflammatory and cardioprotective effects. Biochimie.

[24] Moraes J.C., Coope A., Morari J., Cintra D.E., Roman E.A., Pauli J.R., Romanatto T., Carvalheira J.B., Oliveira A.L.R., Saad M.J. (2009). High-fat diet induces apoptosis of hypothalamic neurons. PLoS ONE.

[25] Menard C., Bastianetto S., Quirion R. (2013). Neuroprotective effects of resveratrol and epigallocatechin gallate polyphenols are mediated by the activation of protein kinase C gamma. Front. Cell Neurosci..

[26] Cintra D.E., Ropelle E.R., Moraes J.C., Pauli J.R., Morari J., de Souza C.T., Grimaldi R., Stahl M., Carvalheira J.B., Saad M.J. (2012). Unsaturated fatty acids revert diet-induced hypothalamic inflammation in obesity. PLoS ONE.

[27] Thaler J.P., Yi C.-X., Schur E.A., Guyenet S.J., Hwang B.H., Dietrich M.O., Zhao X., Sarruf D.A., Izgur V., Maravilla K.R. (2012). Obesity is associated with hypothalamic injury in rodents and humans. J. Clin. Investig..

[28] Milanski M., Degasperi G., Coope A., Morari J., Denis R., Cintra D.E., Tsukumo D.M.L., Anhe G., Amaral M.E., Takahashi H.K. (2009). Saturated fatty acids produce an inflammatory response predominantly through the activation of Tlr4 signaling in hypothalamus: Implications for the pathogenesis of obesity. J. Neurosci..

[29] Viggiano E., Mollica M.P., Lionetti L., Cavaliere G., Trinchese G., De Filippo C., Chieffi S., Gaita M., Barletta A., De Luca B. (2016). Effects of an high-fat diet enriched in lard or in fish oil on the hypothalamic amp-activated protein kinase and inflammatory mediators. Front. Cell. Neurosci..

[30] Araujo E.P., Moraes J.C., Cintra D.E., Velloso L.A. (2016). Mechanisms in endocrinology: Hypothalamic inflammation and nutrition. Eur. J. Endocrinol..

[31] Dragano N.R.V., Marques A.Y.C., Cintra D.E.C., Solon C., Morari J., Leite-Legatti A.V., Velloso L.A., Marósticar M.R. (2013). Freeze-dried jaboticaba peel powder improves insulin sensitivity in high-fat-fed mice. Br. J. Nutr..

[32] Farrell N.J., Norris G.H., Ryan J., Porter C.M., Jiang C., Blesso C.N. (2015). Black elderberry extract attenuates inflammation and metabolic dysfunction in diet-induced obese mice. Br. J. Nutr..

[33] Buettner R., Parhofer K.G., Woenckhaus M., Wrede C.E., Kunz-Schughart L.A., Schölmerich J., Bollheimer L.C. (2006). Defining high-fat-diet rat models: Metabolic and molecular effects of different fat types. J. Mol. Endocrinol..

[34] Blachnio-Zabielska A., Baranowski M., Zabielski P., Gorski J. (2010). Effect of high fat diet enriched with unsaturated and diet rich in saturated fatty acids on sphingolipid metabolism in rat skeletal muscle. J. Cell. Physiol..

[35] Argentato P.P., Morais C.A., Santamarina A.B., de Cassia César H., Estadella D., de Rosso V.V., Pisani L.P. (2017). Jussara (*Euterpe edulis* Mart.) supplementation during pregnancy and lactation modulates UCP-1 and inflammation biomarkers induced by trans-fatty acids in the brown adipose tissue of offspring. Clin. Nutr. Exp..

[36] O’Neill H.M., Holloway G.P., Steinberg G.R. (2013). AMPK regulation of fatty acid metabolism and mitochondrial biogenesis: Implications for obesity. Mol. Cell. Endocrinol..

[37] Luo Z., Ren J., Huang Z., Wang T., Xiang K., Cheng L., Tang L. (2017). The role of exogenous hydrogen sulfide in free fatty acids induced inflammation in macrophages. Cell. Physiol. Biochem..

[38] Riccardi G., Vaccaro O., Costabile G., Rivellese A.A. (2016). How well can we control dyslipidemias through lifestyle modifications?. Curr. Cardiol. Rep..

[39] Qin B., Anderson R.A. (2012). An extract of chokeberry attenuates weight gain and modulates insulin, adipogenic and inflammatory signalling pathways in epididymal adipose tissue of rats fed a fructose-rich diet. Br. J. Nutr..

[40] Mykkänen O.T., Huotari A., Herzig K.H., Dunlop T.W., Mykkänen H., Kirjavainen P.V. (2014). Wild blueberries (vaccinium myrtillus) alleviate inflammation and hypertension associated with developing obesity in mice fed with a high-fat diet. PLoS ONE.

[41] Silva P., Carmo L., Silva G., Silveira-diniz M., Casemiro R., Spoto M. (2013). Physical, Chemical, and Lipid Composition of Juçara (*Euterpe edulis* Mart.) Pulp. Braz. J. Food Nutr..

[42] Karlsen A., Retterstøl L., Laake P., Paur I., Bøhn S.K., Sandvik L., Blomhoff R. (2007). Anthocyanins inhibit nuclear factor-kappaB activation in monocytes and reduce plasma concentrations of pro-inflammatory mediators in healthy adults. J. Nutr..

[43] Qin Y., Xia M., Ma J., Hao Y., Liu J., Mou H., Cao L., Ling W. (2009). Anthocyanin supplementation improves serum LDL-and HDL-cholesterol concentrations associated with the inhibition of cholesteryl ester transfer protein in dyslipidemic subjects. Am. J. Clin. Nutr..

[44] Center for Drug Evaluation and Research (2005). Estimating the maximum safe starting dose in initial clinical trials for therapeutics in adult healthy volunteers. Guidance for Industry.

[45] Graf D., Seifert S., Jaudszus A., Bub A., Watzl B. (2013). Anthocyanin-rich juice lowers serum cholesterol, leptin, and resistin and improves plasma fatty acid composition in fischer rats. PLoS ONE.

[46] Brasil C. (2013). Diretriz Brasileira Para o Cuidado e a Utilização de Animais Para Fins Científicos e Didáticos.

[47] Friedewald W.T., Levy R.I., Fredrickson D.S. (1972). Estimation of the concentration of low-density lipoprotein cholesterol in plasma, without use of the preparative ultracentrifuge. Clin. Chem..

[48] Almeida Morais C., Oyama L.M., de Oliveira J.L., Carvalho Garcia M., de Rosso V.V., Sousa Mendes Amigo L., do Nascimento C.M., Pisani L.P. (2014). Jussara (*Euterpe edulis* Mart.) Supplementation during Pregnancy and Lactation Modulates the Gene and Protein Expression of Inflammation Biomarkers Induced by trans-Fatty Acids in the Colon of Offspring. Med. Inflamm..

[49] Mennitti L.V., Oyama L.M., de Oliveira J.L., Hachul A.C., Santamarina A.B., de Santana A.A., Okuda M.H., Ribeiro E.B., do Nascimento C.M., Pisani L.P. (2014). Oligofructose supplementation during pregnancy and lactation impairs offspring development and alters the intestinal properties of 21-d-old pups. Lipids Health Dis..

